# Cardiac Amyloidosis: A Rare TTR Mutation Found in an Asian Female

**DOI:** 10.3390/jcdd10010013

**Published:** 2023-01-01

**Authors:** Kristina Mouksian, Jessica Ammon, Drenda Pullen, Qiuhua Zhang, Neeraja Yedlapati, John Lynn Jefferies

**Affiliations:** 1 Department of Internal Medicine, Division of Cardiovascular Disease, University of Tennessee Health Science Center, Memphis, TN 38103, USA; 2 Methodist Le Bonheur Healthcare, Memphis, TN 38104, USA; 3 Stern Cardiovascular Foundation, Memphis, TN 38120, USA

**Keywords:** amyloidosis, cardiomyopathy, diastolic heart failure, echocardiography, genetic disorders

## Abstract

Background: Transthyretin cardiac amyloidosis (ATTR) is a life-threatening, debilitating disease caused by abnormal formation and deposit of transthyretin (TTR) protein in multiple tissues, including myocardial extracellular matrix. It can be challenging to diagnose due to the myriad of presenting signs and symptoms. Additionally, numerous TTR mutations exist in certain ethnicities. Interestingly, our patient was discovered to have a very rare Gly67Ala TTR mutation typically not found in individuals of Asian descent. Recent advances in cardiovascular imaging techniques have allowed for earlier recognition and, therefore, management of this disease. Although incurable, there are now new, emerging treatments that are available for symptom control of cardiac amyloidosis, making early diagnosis vital for these patients, specifically their quality of life. Case summary: We outline a case of a 50-year-old Asian female who was initially hospitalized for nausea and vomiting and persistent orthostatic hypotension. She underwent a multitude of laboratory and imaging tests, resulting in a diagnosis of cardiac amyloidosis, which was confirmed to be due to a rare TTR mutation via genetic testing. Conclusions: Our objective is to describe various TTR mutations, existing diagnostic imaging modalities, and available treatments, as well as highlight the importance of early screening and awareness of cardiac amyloidosis, allowing for quicker diagnosis and treatment of this disease.

## 1. Introduction

Diagnosing cardiac amyloidosis can be challenging because of the numerous vague signs and symptoms that could be attributed to other diseases. Additionally, there are different genetic mutations in TTR genes that exist based on ethnicity. Fortunately, due to advances in cardiovascular imaging techniques and genetic testing, previously undiagnosed and uncommon conditions, such as cardiac amyloidosis, are being increasingly identified. There are now several medications available for management of TTR amyloidosis that limit further amyloid deposition, thus, altering the disease course. In this case report, we discuss a 50-year-old Asian female who was admitted with vague symptoms with subsequent workup confirming the diagnosis of TTR amyloidosis. A review of TTR amyloidosis focusing on symptoms, diagnosis, and treatment is also included.

## 2. Case Presentation

### 2.1. Chief Complaints

A 50-year-old Asian female presented to the hospital with dysphagia, diarrhea, retractable nausea and vomiting, and persistent orthostatic hypotension. She was admitted for further workup due to debilitating orthostatic hypotension and dehydration.

### 2.2. History of Past Illness

Her medical history was notable for gastrointestinal amyloidosis (diagnosed via stomach biopsy; unknown mutation due to lack of records), persistent nausea, peripheral neuropathy, and carpal tunnel syndrome. However, due to the patient recently immigrating to the United States from Vietnam, it was difficult to ascertain complete medical history without any available documentation from her doctors in her home country.

### 2.3. Medication History

Prior to the diagnosis of cardiac amyloidosis, the patient was taking 40 mg pantoprazole twice daily for acid reflux symptoms and metoclopramide as needed for nausea.

### 2.4. Physical Exam and Review of Systems upon Admission

On initial physical examination, she was afebrile with a blood pressure of 86/73, heart rate of 88 beats/min, respiratory rate of 18 breaths/min, and pulse oximetry saturation of 97% on room air. Her chest was clear to auscultation; she had a regular rhythm and no audible murmurs; her jugular venous pressure was not elevated; her abdomen was non-tender to palpation. She had good pulses bilaterally in both the upper and lower extremities. She did not complain of fever, headaches, vision changes, myalgias, fatigue, chest pain, palpitations, skipped beats, shortness of breath, or edema. She later did endorse bilateral upper and lower extremity paresthesia, syncopal episodes, and dizziness in addition to her presenting complaints.

### 2.5. Medical Management

On initial presentation at the hospital, she was volume resuscitated with intravenous fluids without improvement in orthostasis. Midodrine and fludrocortisone were started, as well as antiemetics and proton pump inhibitors for symptom relief. With improvement in her hemodynamic status, she was able to be discharged and followed up in our cardiomyopathy clinic for further management.

### 2.6. Laboratory Findings

Routine blood tests and cardiac biomarkers were obtained (Table 1). To first rule out gammopathy, serum and urine electrophoresis, serum-free light chains were obtained and were negative for Amyloidosis light chain (AL).

### 2.7. Imaging Examination

**Figure 1:**
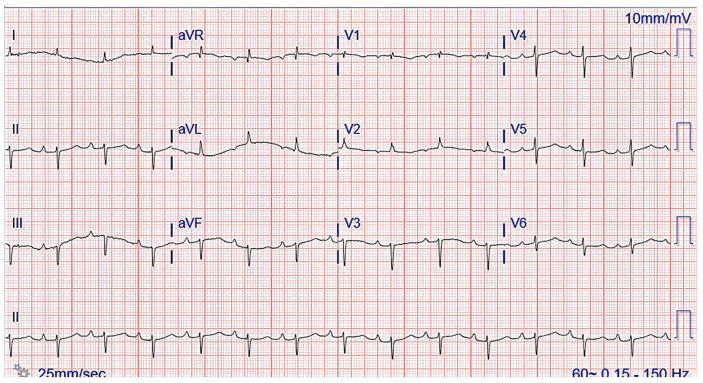
ECG showing normal sinus rhythm with first-degree atrioventricular block and left axis deviation.

**Figure 2:**
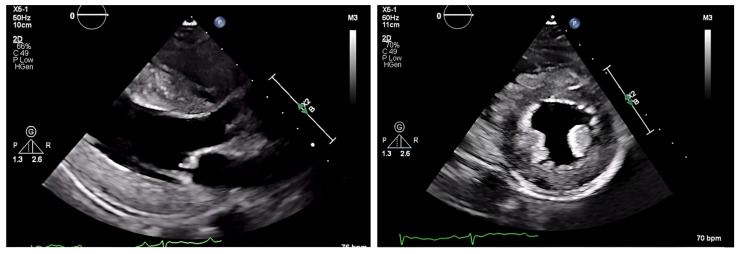
ECHO showing a discrepancy between the wall mass and electrical activity on ECG.

**Figure 3:**
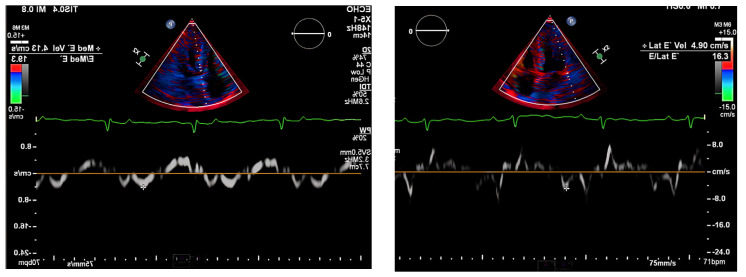
Pulse-wave Tissue Doppler ECHO showing elevated E/E′ ratio and low mitral annulus velocities suggestive of diastolic dysfunction.

The chest radiograph did not show any acute findings. Electrocardiogram (ECG) (Figure 1) showed normal sinus rhythm with first-degree atrioventricular block, left axis deviation, and low voltage in limb leads. No prior echocardiogram (ECHO) was available, so an initial ECHO was obtained showing preserved ventricular ejection fraction (EF), moderate left ventricular hypertrophy (LVH), stage II diastolic dysfunction, and small pericardial effusion (Figure 2 and Figure 3).

Owing to the complexity and the multisystem involvement, along with the patient’s symptoms and history of GI amyloidosis, the main differential diagnosis was cardiac amyloidosis. Other differentials, such as high-output heart failure and restrictive cardiomyopathy, were considered but were less consistent with the patient’s presentation.

**Figure 4:**
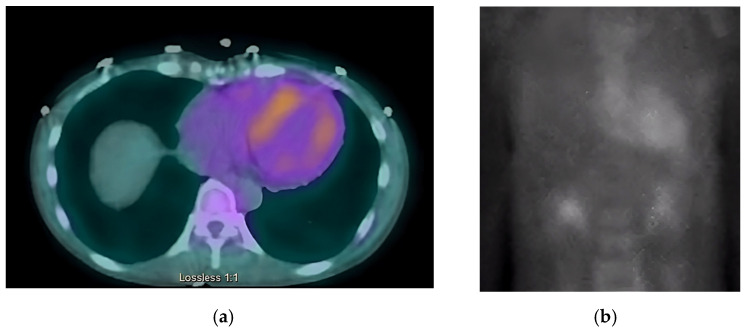
SPECT in an ATTR cardiac amyloidosis patient. Evidence of uptake of ^99m^Tc-PYP in the myocardium. (**a**) Quantitative SPECT/CT; (**b**) Semiquantitative analysis of ^99m^Tc-PYP myocardial uptake.

**Figure 5:**
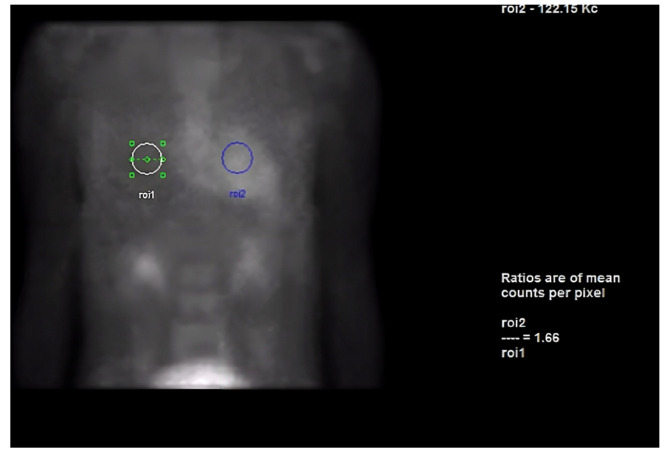
Heart-to-contralateral ratio was calculated by drawing a region of interest over the heart, mirroring it to the contralateral chest, and calculating the ratio of heart ROI to contralateral chest ROI mean counts. The representative image demonstrates a H/CL ratio of 1.6. (H/CL, heart-to-contralateral; ROI, region of interest).

Next, we performed a ^99^Technetium pyrophosphate (^99m^Tc-PYP) planar scintigraphy (Figure 4). A quantitative analysis of the heart retention pattern showed a heart-to-contralateral lung (H/Cl) ratio of 1.66 (Figure 5), strongly suggestive of amyloid deposition. To differentiate the type of cardiac amyloidosis, genetic testing was obtained and revealed a heterozygous pathogenic mutation in the TTR gene, diagnostic for TTR amyloidosis.

Of note, the patient does have a strong family history of amyloidosis. She has stated that four out of her seven siblings have passed away from amyloidosis in Vietnam (three brothers and one sister). It is unknown if the patient’s parents also suffered from this disease. Unfortunately, family history cannot be verified, and therefore, a pedigree chart cannot be completed. Cascade screening performed for her two sons living in the United States was found to be negative.

### 2.8. Final Diagnosis

Hereditary transthyretin cardiac amyloidosis secondary to Gly67Ala mutation.

### 2.9. Treatment

The main goal of her treatment was to optimize her cardiac function and symptom management. Our patient had numerous manifestations of both cardiac and GI amyloidosis that affected her daily life. She underwent multiple esophagogastroduodenoscopies (EGD) for her ongoing dysphagia, complaint of food being stuck in her throat, as well as persistent nausea and vomiting. Initially, she was diagnosed with reflux esophagitis, but with repeat imaging studies, she was eventually also diagnosed with gastroparesis, for which she was continued on metoclopramide and pantoprazole. Her orthostatic hypotension was treated with 2.5 mg midodrine three times daily and 0.1 mg fludrocortisone daily. She was sent to a neurologist for further evaluation of her peripheral neuropathy. She underwent nerve conduction and EMG studies and was found to have moderate axonal sensorimotor polyneuropathy with superimposed bilateral median nerve neuropathy. She was initially prescribed gabapentin with mild relief of her symptoms, but it was discontinued due to the patient’s persistent syncopal episodes. She was then started on TTR-specific medication 284 mcg/1.5 mL Inotersen subcutaneously weekly for her neuropathic symptoms related to TTR amyloidosis. Although Inotersen somewhat reduced her symptom of neuropathy, it was eventually discontinued due to persistent thrombocytopenia, and unfortunately, Tafamidis was not approved at that time. She was later started on Patisiran infusion, at which point she was finally able to stand up with minimal dizziness. However, approximately six months after the initiation of Patisiran, it too had to be stopped due to the patient’s intolerance to this medication, with the main complaints of debilitating fatigue and persistent nausea and vomiting. At that time, the patient did agree to start Tafamadis, and ongoing efforts are being made to make this drug available to this patient.

### 2.10. Outcome and Follow-Up

Her overall condition and response to treatment were closely monitored in our clinic with monthly follow-up visits. The patient initially tolerated medications as stated above. However, despite medical management, her overall condition has been deteriorating. She continued to have a poor appetite and lost a significant amount of weight which further contributed to her weakness and fatigue. She has been hospitalized a multitude of times throughout the last two years with ongoing dysphagia, hypotension, orthostasis, syncope, nausea, and vomiting, ultimately being admitted for dehydration and malnutrition. The patient eventually underwent further evaluation by her gastroenterologist, and a percutaneous endoscopic gastrostomy tube was placed for nutrition purposes. She continues to follow up with cardiology and gastroenterology clinics. She was also hospitalized on several occasions with obstructive urinary tract infections and urinary retention, which ultimately resulted in the placement of a chronic foley catheter. She is now being closely monitored by urology services as well. The patient had several repeat ECHOs without major changes from imaging obtained on the initial clinic presentation. The most recent ECHO is without any significant changes. Her EF continues to be normal, and she continues to have moderate LVH and stage II diastolic dysfunction with persistent, small pericardial effusion. Fortunately, she was never hospitalized with symptoms of heart failure. Her current medication regimen includes pantoprazole, amitriptyline, gabapentin, fludrocortisone, midodrine, droxidopa, and metoclopramide. Her main complaints remain to be orthostatic hypotension, syncope, dizziness, weakness, and peripheral neuropathy with resultant insomnia. 

## 3. Discussion

Amyloidosis is a rare disease characterized by extracellular tissue deposition of insoluble fibrils composed of abnormally folded proteins [1,2]. Protein folding is a complex process in which a linear polypeptide chain folds, usually into α-helices, ultimately reaching a 3D structure. Multiple factors can disrupt this process and lead to proteins that misfold and aggregate. Examples include mutations, posttranslational modifications, and exogenous factors such as thermal stress. Cell quality control systems focused on proteostasis include molecular chaperones, the ubiquitin-proteasome system, and autophagy. When these systems reach capacity, there is an increased propensity for protein misfolding and aggregation. Amyloid fibrils formed may remain localized to their production site or circulate and deposit systemically, depending on the etiology of the amyloidosis [3,4]. Amyloid deposits can be visualized with polarized light microscopy. They demonstrate apple-green birefringence with Congo red staining and have nonbranching, rigid filaments typically 7.5–10 nanometers in diameter [4].

Amyloidosis can be hereditary or acquired. There are several types, and the classification is based on the specific amyloid protein comprising the deposited fibrils [1,5]. Currently, at least 36 different human protein precursors of amyloid fibrils exist, but more than 60 heterogeneous amyloidogenic proteins have been identified [6]. A shared characteristic of these misfolded proteins is their propensity to form β-pleated sheets with antiparallel alignment, which contributes to their rigidity and proteolytic-resistant structure. Concerning cardiac amyloidosis (CA), two types are responsible for approximately 95% of cases: Immunoglobulin light chain amyloidosis (AL) and transthyretin amyloidosis (ATTR) [6,7,8,9]. This review will emphasize ATTR with a brief mention of AL below. 

Immunoglobulin light chain amyloidosis is the most common and severe type of systemic amyloidosis. It occurs when an abnormal clone of plasma cells overproduces free light chains normally associated with immunoglobulins. The circulating free light chains are digested with pepsin to form amyloid fibrils that deposit systemically with subsequent clinical manifestation [3,9,10]. It is a multiorgan disease that most commonly affects the kidney, although cardiac manifestations are the second most common presentation. AL can occur independently or with other monoclonal gammopathies (e.g., multiple myeloma, monoclonal gammopathy of undetermined significance, Waldenstrom macroglobulinemia, etc.) [8,9,10,11]. It is typically seen in patients >50 years old, with a male predominance [7,9]. Early diagnosis and treatment are important to alter the outcome of the disease.

Transthyretin amyloidosis results from misfolded transthyretin proteins that aggregate, forming amyloid fibrils that deposit into organs. Transthyretin is a transport protein synthesized in the liver, retina, and choroid plexus. It primarily circulates as a tetramer in blood and cerebrospinal fluid. Normal transthyretin proteins form a tetramer to transport thyroxine and, with the addition of a retinol-binding protein, can transport retinol (Vitamin A) [12]. ATTR has two main types: hereditary transthyretin amyloidosis (hATTR) and wild-type transthyretin amyloidosis (wtATTR). hATTR is caused by genetic mutations of the TTR gene on chromosome 18. TTR mutations destabilize the tetramer leading to misfolding and eventual amyloid fibril formation that deposits in multiple tissues, mainly the heart and peripheral nervous system. In wtATTR, transthyretin tetramer dissociation occurs sporadically, though the aging process may contribute to this [13].

**Figure 6:**
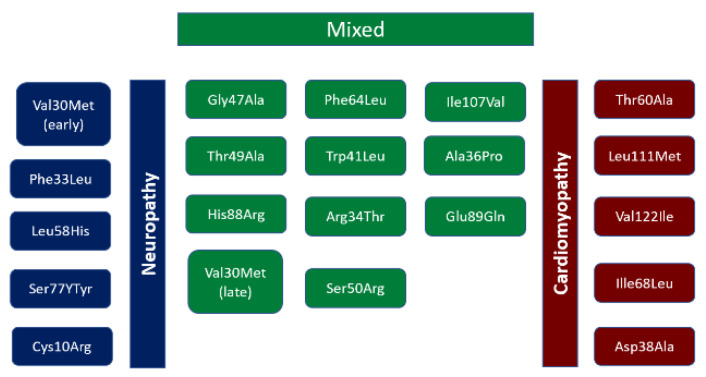
Spectrum of TTR mutations and their phenotypic features. Adapted from Semigran M., J. [22].

The phenotype of hATTR-CM depends on a specific mutation of the gene. It is inherited in an autosomal dominant fashion with more than 120 such known mutations involving the TTR gene, and these differ based on geographic and ethnic groups (Table 2). Manifestations of this disease are dependent on specific mutation present (Figure 6). Previously, three major endemic clusters have been described located in Portugal, Sweden, and Japan, with smaller clusters in Cyprus and Majorca. Europe has had a sporadic incidence of disease [12]. The most common mutation worldwide is Val30Met (Valine to Methionine at position 30), which is responsible for Familial Autonomic Neuropathy, FAP, type 1. It is endemic to Portugal, Sweden, and Japan. Early onset patients (<50 years old) typically present with sensory-motor and autonomic neuropathy, while cardiac and ocular involvement increases with disease duration. From a cardiac standpoint, mutations of particular significance are Val122Ile (Valine to Isoleucine at position 122) found in the United States, Thr60Ala (Threonine to Alanine at position 60) in the United Kingdom, and Ile68Leu (sIsoleucine to Leucine at position 68) in Italy [8]. The Val122Ile is the most common hATTR subtype in the US, found in approximately 4% of the African American population. However, this may be underestimated due to healthcare disparities and underdiagnosis of CA. The penetrance of the Val122Ile mutation is unknown. The Val122Ile subtype has a predominantly cardiac phenotype, but if other manifestations are present, it is most commonly carpal tunnel syndrome and spinal stenosis [8,14,15]. Leu111Met (Leucine to Methionine at position 11) and Ile68Leu (Isoleucine to Leucine at position 68) are endemic to Denmark and Italy. Asp38Ala (Aspartate to Alanine at position 38) is the most common mutation found in Japan [16].

wtATTR has a predominant cardiac phenotype and is the most common cause of CA in the US. It occurs sporadically, and most patients with wtATTR CA are >65 years old with a male and Caucasian predominance. Carpal tunnel syndrome is a common feature in wtATTR (~50% of patients) and usually precedes cardiomyopathy symptoms by 5-10 years [13,17]. Our patient’s mutation of Gly67Ala is very rare and has previously only been documented in German Italian, and Mediterranean populations [18]. Unfortunately, data regarding Gly67Ala is scarce. Small studies in Mexican and French patients with Gly47Ala mutation showed a clinical predominance of neuropathy [19,20]. Literature on TTR-related cardiac amyloidosis in Asian populations is limited and is exclusively case series. One such study [21] demonstrated the genotypic and phenotypic characteristics of familial TTR amyloidosis in a Southeast Asian cohort, where out of 29 patients from China, Malaysia, Myanmar, Vietnam, and Indonesia, 5 patients were found to have early-onset disease (age <50 years) with multiple variants which included Gly47Ala mutation. They have found somatic neuropathy to be the most common initial symptom, followed by carpal tunnel syndrome, as well as autonomic and cardiac dysfunction. More studies are needed to assess the incidence and prevalence of this disease in Asians around the world.

### 3.1. Pathophysiology

Amyloid fibrils can deposit throughout the heart, including valves, endocardium, myocardial interstitium, epicardium, parietal pericardium, and coronary arteries (specifically the vasa vasorum of epicardial coronary arteries and small intramural coronary branches) [23]. The diffuse deposition of amyloid in the cardiac interstitial space leads to cardiomyocyte necrosis and interstitial fibrosis, resulting in biventricular wall thickening and stiffening, leading to impaired diastolic relaxation. These are trademarks of this restrictive cardiomyopathy [24,25]. In CA, atrial assessment has historically focused on atrial dimensions, not atrial function. Chronicity and advanced diastolic dysfunction of the disease contributes to left atrial (LA) dilation from the high left ventricle (LV) filling pressures. Atria, valves, and conduction system involvement can lead to atrial fibrillation (AF), valvular dysfunction, and various degrees of heart block. Fibril deposition within the coronary arteries can be non-obstructive or obstructive, causing ischemia/infarct [26].

### 3.2. Clinical Manifestations

CA results in a restrictive cardiomyopathy with diastolic dysfunction—heart failure with preserved ejection fraction (HFpEF). Patients typically present with dyspnea, orthopnea, and right-sided heart failure symptoms, including lower extremity edema, hepatomegaly, ascites, and elevated jugular venous pressure [8]. Fatigue, weakness, diminished pulse pressure and decreased capillary refill can also occur from low cardiac output (CO) [27]. Conduction system involvement can cause varying degrees of heart block, presenting with fatigue, syncope, or sudden cardiac arrest. The most common arrhythmia in this population is AF occurring in approximately 10–20% of patients and increasing their risk of thromboembolism [28]. Angina or infarction can occur with coronary artery involvement. Small pericardial effusions can happen, but it is relatively rare to have a large effusion or cardiac tamponade. Extracardiac manifestations include macroglossia, periorbital ecchymosis, purpura, constipation, diarrhea, fatigue, weight loss, neuropathy (peripheral and autonomic), bilateral carpal tunnel syndrome, and spinal stenosis [25]. Our patient had multiple signs and symptoms suggestive of cardiac amyloidosis that helped us achieve a quick diagnosis, including autonomic dysfunction (peripheral neuropathy, dizziness, syncope, urinary retention), HFpEF with diastolic dysfunction, first-degree AVB, small, persistent pericardial effusion, and bilateral carpal tunnel syndrome. It is critical for clinicians to be aware of common disease patterns and to recognize diagnostic signs (Table 3).

### 3.3. Clinical Evaluation

ATTR amyloidosis is not a rare disease but rather underdiagnosed due to vague, multisystem involvement and common signs and symptoms that mislead clinicians to diagnose patients with HFpEF. In fact, cardiac amyloidosis is thought to be underdiagnosed in a significant proportion of patients with HFpEF [29]. Overall survival is poor once there is cardiac involvement. The key to diagnosis is a high index of suspicion, at which point a rapid, targeted diagnostic evaluation and initiation of treatment should be performed to improve the prognosis, all-cause mortality, and hospitalization of these patients [30]. There has been an area of advancement to aid in the diagnosis and treatment of ATTR-CM.

**Figure 7:**
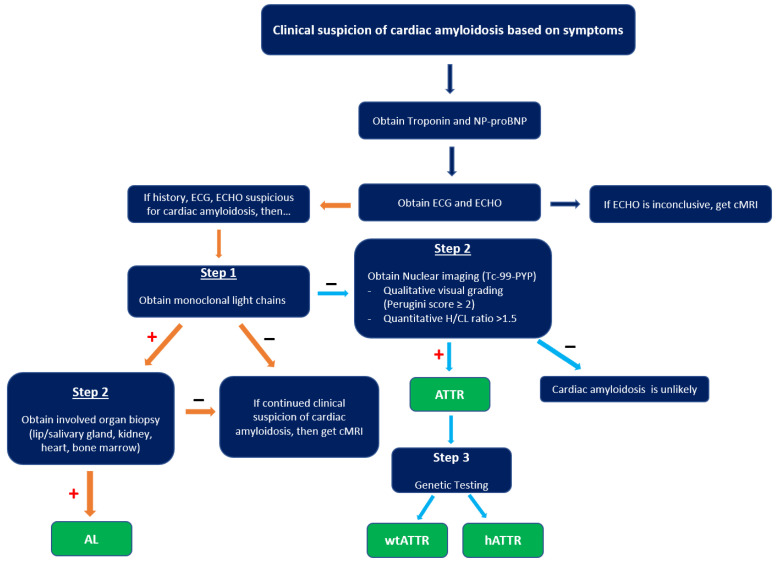
Diagnostic algorithm of cardiac amyloidosis. cMRI, cardiac magnetic resonance imaging; H/CL, heart-to-contralateral lung ratio; AL, amyloid light chain; wtATTR, transthyretin amyloid wild type; hATTR, transthyretin amyloid hereditary. “+” indicates criteria met in prior box, while “–“ indicates criteria not met in prior box.

When suspicious for CA, a typical evaluation includes labs, ECG, imaging, and possibly histopathology (Figure 7).

The plasma level of brain natriuretic peptide (BNP) and N-terminal proBNP (NT-proBNP) are increased in heart failure but may be disproportionately increased in CA from cardiomyocyte compression and elevated cardiac filling pressure. Troponin T, troponin I, or both may be chronically elevated from the ongoing myocardial damage occurring in CA [5,31]. In the initial workup of CA, evaluation for AL is indicated, including serum protein electrophoresis with immunofixation (SPEP), urine protein electrophoresis with immunofixation (UPEP), and serum free light chain (FLC) assay. SPEP and UPEP evaluate clonal immunoglobulin and light chain production, respectively. 

One of the first clues of suspected CA is the EKG. A characteristic ECG finding in CA is low voltage QRS in all limb leads (as amyloid fibrils are electrically silent) and poor R wave progression or a pseudoinfarct pattern with Q waves in precordial leads [32]. This is incongruent with the typical finding of ventricular thickening seen on echocardiography (ECHO). However, with only 30–70% of patients meeting low voltage criteria on EKG, its absence cannot preclude a diagnosis of CA [2]. Other common ECG findings are AF, intraventricular conduction delays, various atrioventricular blocks, and bundle branch blocks. 

On ECHO, the characteristic finding is symmetric biventricular wall thickening (LV > 12 mm, and often ≥15 mm) with non-dilated ventricles and diastolic dysfunction. Biatrial enlargement commonly occurs from elevated ventricular filling pressure and direct atrial amyloid deposition. There is a risk for atrial thrombus formation (in the absence of arrhythmias) as amyloid infiltration contributes to an irregular endocardial surface, decreased atrial contractility, and decreased stroke volume (SV) [33].

Valvular thickening may be present and can help differentiate CA from hypertensive heart disease [27]. EF is initially preserved with systolic dysfunction absent until advanced stages are reached when sequelae of myocyte necrosis and fibrosis start affecting systolic function. A hyperechoic “granular sparkling” appearing myocardium may be present. 2D speckle ECHO allows for analysis of the longitudinal axis. Impaired longitudinal strain in the basal and midventricular wall with sparing of the apical region is a characteristic finding. This finding has a high sensitivity rate (90–95%) and a high specificity rate (80–85%) [28]. It is also common to see small pericardial effusions, though this is nonspecific.

Radionuclide bone scintigraphy uses a bone-avid, phosphate-based isotope (^99m^Tc-PYP) as a noninvasive way to diagnose ATTR. Phosphate-calcium binding seems to play an important role in the myocardial uptake of tracers, though the exact mechanism is unknown. Increased tracer accumulation in ATTR may suggest it has a higher calcium level than AL, and/or the duration of amyloid deposition increases uptake as ATTR has a more indolent course. Scoring cardiac uptake of the tracer is done via a semi-quantitative method using the Perugini grading scale (graded from 0–3). It visually compares tracer uptake in the myocardium and ribs of the planar image. ^99m^Tc-PYP with abnormal uptake (grade 2 or 3) in the absence of a monoclonal gammopathy has a specificity and positive predictive value of 100% for diagnosing ATTR-CA. However, genotyping is still required to differentiate hATTR and wtATTR. If hATTR is present, first-degree relatives should be tested for the identified variant [25]. A heart-to-contralateral lung ratio > 1.5 has a 97% sensitivity and 100% specificity in differentiating ATTR from AL [28]. In patients with a monoclonal gammopathy, scintigraphy alone cannot be used to diagnose ATTR, and an endomyocardial biopsy (EMB) is required for diagnosis. Currently, imaging techniques allow for an accurate and noninvasive method to diagnose ATTR without the need for confirmatory myocardial biopsy. However, if you need to differentiate ATTR with concomitant MGUS from AL, an EMB is indicated. Lastly, a plasma cell dyscrasia with equivocal cardiac imaging findings is another indication for EMB [28].

### 3.4. Treatment

Recent developments of multiple medications have been proven beneficial in this patient population. The main goal of treatment is optimizing cardiac function and symptom management. In ATTR, disease-modifying therapies act on various steps in the ATTRA-CA amyloid production process. The two main approaches to treating amyloidosis are stopping the production of precursor protein and reducing the burden of pre-existing amyloid deposition. There are several drugs currently available for the management of ATTR. TTR silencers, such as *Inotersen* and *Patisiran,* suppress hepatic TTR mRNA resulting in reduced production of TTR amyloid. TTR stabilizers, such as *Tafadimis* and *Diflunisal,* bind to TTR tetramer, therefore, inhibiting its dissociation into monomers, resulting in a reduction of amyloid formation. ATTR degraders like doxycycline with TUDCA/UDCA (tauroursodeoxycholic acid/ursodeoxycholic acid) promote the breakdown and clearance of amyloid fibrils. Additionally, certain hATTR patients are eligible for liver transplants as this is the primary production site of the amyloidogenic protein [11,34]. These therapies have been proven to be more effective when initiated prior to cardiac involvement. Therefore, early identification of ATTR-CM is essential. 

Supportive treatment for heart failure symptoms and arrhythmias is available. In CA, the ventricles are very stiff, and SV is reduced; therefore, even small volume changes can have profound hemodynamic effects. For patients with symptoms of volume overload, loop diuretics and mineralocorticoid receptor antagonists are used. Additionally, a low-sodium diet and daily weights are recommended. Most guideline directed medical therapy (GDMT) is poorly tolerated and/or not studied in patients with HF from CA. Angiotensin-converting enzyme inhibitors, angiotensin receptor blockers, and angiotensin receptor/neprilysin inhibitors provoke severe hypotension in hATTR, likely due to subclinical autonomic neuropathy. They may be better tolerated in wtATTR, where autonomic neuropathy is rarely involved; however, there have been no clinical trials of these medications in CA patients. Βeta-blockers are poorly tolerated in patients with HF from CA due to diminished SV at baseline with a CO that is dependent on their heart rate. Non-dihydropyridine calcium channel blockers are contraindicated in HF from CA due to negative inotropic effects and a decrease in HR. Digoxin should not be used for arrhythmias in CA patients as it strongly binds to amyloid fibrils, increasing toxicity risk. If AF occurs, amiodarone is recommended for rhythm control. CA has an increased risk for intracardiac thrombus formation; if a CA patient develops AF, anticoagulation is indicated regardless of the CHA_2_DS_2_-VASc score. A permanent pacemaker may be indicated in CA patients with symptomatic or severe AV block. Sudden cardiac death is not uncommon in this patient population and is typically from tachyarrhythmias, pulseless electrical activity, or AV block in advanced stages of cardiomyopathy [35]. In patients with CA who are resuscitated from a life-threatening ventricular arrhythmia, ICD implantation should be considered. However, there is limited data to support ICD use in primary prevention in CA patients [34,35].

## 4. Conclusions

Cardiac amyloidosis is challenging to diagnose and treat. Recent advancements in cardiac imaging and the emergence of medications allow for early identification and treatment and result in improved clinical outcomes. Our case report discusses the clinical evaluation of cardiac amyloidosis, highlighting the importance of recognizing diagnostic clues, utilization of diagnostic steps, the importance of early screening, and awareness of unique TTR mutations that may occur in different ethnic populations, which is imperative for prompt diagnosis and treatment of these patients. However, further studies in Asian populations are needed for better recognition of this debilitating disease in that population.

## Figures and Tables

**Table 1:** Laboratory tests.

	Results	Normal Range
**White blood cell count (K/u/L)**	5.2 K/uL	4–11
**Hemoglobin (g/dL)**	13.4g/dL	12–16
**Platelets (K/u/L)**	206 K/uL	150–450
**Urea mg/dL**	6 mg/dL	6–20
**Creatinine mg/dL**	0.54 mg/dL	0.60–1.30
**Pro-B-type natriuretic peptide (pg/L)**	347 pg/L	<300
**Thyroid stimulating hormone mlU/L**	2.5 mlU/L	0.40–4.1
**Hemoglobin A1C %**	5.6%	4.2–5.6
**Serum and urine electrophoresis**	Negative for light chain	

**Table 2:** Specific TTR mutations and epidemiology [12].

TTR Mutation	Typical Location
Val30Met	Portugal, Brazil, Japan, Sweden, France, Italy, USA
Val122Ile	USA
Thr60Ala	UK, USA
Leu111Met, Ille68Leu	Denmark, Italy, Japan
Asp8Ala	Japan
Ser50Arg	Japan, France, Italy, USA
Ser77Tyr	USA, France, Israel
Ile107Val	USA, France, Brazil
Gly67Ala	Germany, Italy, Mexico, France, China, Malaysia, Myanmar, Vietnam, Indonesia

**Table 3:** Diagnostic clues of ATTR-CM.

History, Examination, and Labs	Evidence of Right-Sided Heart Failure (Ascites, Lower Extremity Edema, etc.)
	HFpEF
	Intolerance to ACEi and BB
	* Chronic elevation of troponin and BNP
	* Bilateral carpal tunnel syndrome
	Lumbar stenosis
	* Unexplained peripheral neuropathy
	* Autonomic dysfunction
	Arrhythmias or conduction system delays
	* Low voltage EKG
Imaging	Myocardial uptake on PYP imaging
	ECHO with biventricular hypertrophy, valve thickening, atrial septal thickening
	Apical sparing on longitudinal strain imaging

* Indicates red flag signs, ACEi = angiotensin converting enzyme inhibitor, BB = beta blocker, HFpEF = heart failure with preserved ejection fraction.

## Data Availability

Not applicable.

## Abbreviations

ECG	electrocardiogram
ECHO	echocardiogram
^99m^Tc-PYP	technicium-99 pyrophosphate
ATTR	transthyretin amyloidosis
CM	cardiomyopathy
HF	heart failure
AL	light chain amyloidosis
CA	cardiac amyloidosis
SV	stroke volume
CO	cardiac output
hATTR	hereditary TTR amyloidosis
wtATTR	wild type TTR amyloidosis
